# Microfluidic Encapsulation of Sorafenib-Loaded ZIF‑8 Nanoparticles in pH-Responsive Alginate Microparticles for Oral Chemotherapy of Hepatocellular Carcinoma

**DOI:** 10.1021/acsabm.5c01270

**Published:** 2026-01-28

**Authors:** Mojdeh Mirshafiei, Zahra Mahmoudi, Mehdi Mehrpouya, Mahdi Mahmoudi, Masoud Rezaeian, Mona Navaei-Nigjeh, Zahra Katoli, Lobat Tayebi

**Affiliations:** † Department of Biotechnology, School of Chemical Engineering, College of Engineering, 48425 University of Tehran, Tehran 143951561, Iran; ‡ Department of Medical Biotechnology, School of Biotechnology, College of Science, 48425 University of Tehran, Tehran 6515744949, Iran; § School of Energy Engineering and Sustainable Resources, College of Interdisciplinary Science and Technology, 48425 University of Tehran, Tehran 1431895648, Iran; ∥ Hydrogen and Fuel Cell Laboratory, College of Interdisciplinary Science and Technology, 48425 University of Tehran, Tehran 1431895648, Iran; ⊥ Department of Chemical and Biological Engineering, The University of British Columbia, 2360 E Mall, Vancouver, British Columbia V6T 1Z3, Canada; # Pharmaceutical Science Research Center (PSRC), Tehran University of Medical Science (TUMS), Tehran 1416753955, Iran; ∇ Department of Pharmaceutical Biomaterials and Medical Biomaterials Research Center, Faculty of Pharmacy, Tehran University of Medical Science (TUMS), Tehran 1416753955, Iran; ○ Department of Life Science Engineering, Faculty of New Science & Technologies, 48425 University of Tehran, Tehran 1439957131, Iran; ◆ Institute for Engineering in Medicine, Health, & Human Performance (EnMed), Batten College of Engineering and Technology, Old Dominion University, Norfolk, Virginia 23529, United States

**Keywords:** Alginate microparticles, Microfluidics, Nano-in-microparticles, pH-responsive, ZIF-8 nanoparticles (NPs), Oral drug delivery

## Abstract

Hepatocellular carcinoma (HCC) remains one of the leading causes of cancer-related mortality. Sorafenib is the current first-line oral therapy; however, its therapeutic efficacy is limited by poor aqueous solubility, low bioavailability, and gastrointestinal instability. This study aimed to develop a pH-responsive nano-in-microparticle delivery system using a single-step droplet-based microfluidic process to protect sorafenib in the gastric environment and achieve controlled release for enhanced oral chemotherapy. Sorafenib-loaded ZIF-8 nanoparticles (SZ NPs) were synthesized and characterized by scanning electron microscopy (SEM), Fourier-transform infrared (FTIR) spectroscopy, Energy-dispersive X-ray (EDX) spectroscopy, and X-ray diffraction (XRD), exhibiting a mean diameter of about 72 nm and a drug encapsulation efficiency of 76%. These SZ NPs were subsequently encapsulated in alginate to form pH-responsive nano-in-microparticles. Computational fluid dynamics (CFD) simulations were conducted to optimize flow dynamics and droplet formation within the microfluidic channels, and particle morphology and uniformity were assessed via bright-field microscopy, fluorescence microscopy, and SEM. The resulting nano-in-microparticles exhibited spherical morphology with hydrodynamic sizes ranging from 76 to 113 μm, depending on the flow rate ratio, demonstrating uniform SZ NP dispersion. Drug release studies in simulated gastric and intestinal fluids revealed that the nano-in-microparticles prevented premature drug release in acidic simulated gastric fluid (pH < 5.7), while facilitating a controlled and sustained release profile under simulated intestinal conditions (pH 7.4) over 24 h. Cytotoxicity assays against HepG2 liver cancer cells showed significant anticancer efficacy compared to free sorafenib. These findings highlight the potential of this pH-responsive platform as an effective oral delivery strategy for HCC therapy.

## Graphical abstract

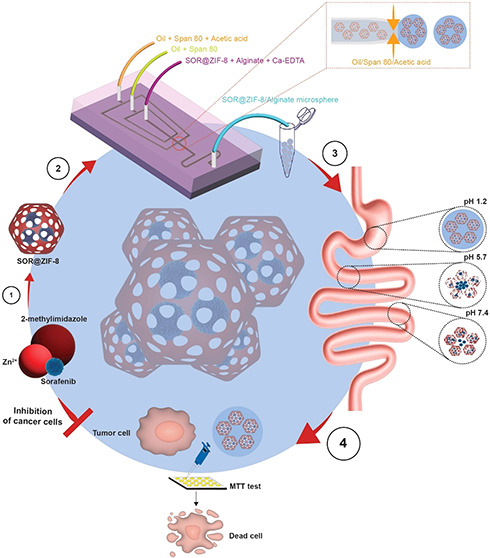

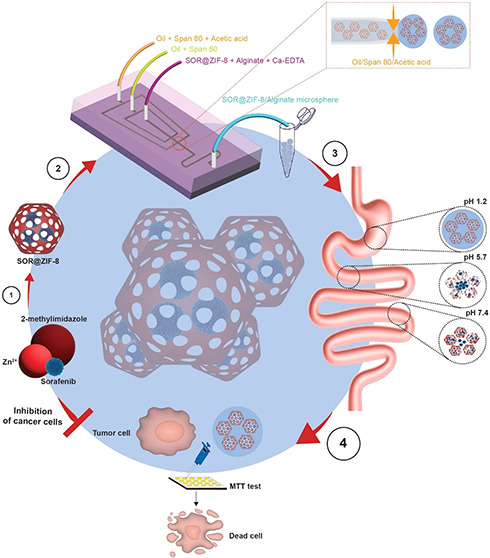

## 1 Introduction

Hepatocellular carcinoma (HCC) is the most prevalent form of liver cancer and the sixth most commonly diagnosed cancer.^1^ Sorafenib has been widely utilized in the therapeutic management of HCC. Nevertheless, the hydrophobic nature of this drug and its low solubility in the blood circulation result in limited oral bioavailability, hindering its optimal efficacy and therapeutic applications.^2^ Recent advances in nanobased drug delivery systems have sought to overcome the limitations of conventional chemotherapeutics and enhance their therapeutic outcomes.^3^ Zeolitic Imidazolate Framework-8 (ZIF-8), a subclass of metal–organic frameworks (MOFs), has garnered considerable attention for smart drug delivery systems due to its promising characteristics, such as drug loading capabilities, biocompatibility, and low toxicity.^4,5^ Its pH-responsive nature allows controlled cargo release upon facing low acidic pH environments, encouraging many to recruit ZIF-8 NPs for the delivery of hydrophobic drugs to the tumor site.^6,7^ The route of oral drug delivery has manifested promising outcomes, offering a convenient and patient-friendly approach for various types of nanotherapeutics.^8^ Nevertheless, the instability of ZIF-8 in the acidic gastric environment often leads to premature degradation and burst release of encapsulated drugs, thereby limiting its effectiveness in oral formulations.^9^ Consequently, effective oral delivery of sorafenib-loaded ZIF-8 nanoparticles (SZ NPs) requires protection against the acidic gastric environment to ensure their successful delivery to the intestinal tract for absorption.^10,11^ To circumvent this, protective strategies such as surface modification with phospholipids, tannic acid, or targeting ligands such as RGD peptides have been investigated for efficient delivery of ZIF-8 NPs to the tumor site.^7,12^ Another solution to overcome this challenge is integrating NPs with pH-responsive polymers to achieve controlled release under specific pH conditions.^13,14^

Among various pH-sensitive polymers, alginate has attracted particular attention for oral drug delivery applications. Alginate undergoes protonation of its carboxyl groups in acidic media, forming a compact gel structure that limits drug diffusion, while at higher pH values (≥5.5), deprotonation induces swelling and facilitates controlled release.^15–17^ Owing to its biocompatibility, biodegradability, and nonimmunogenic nature, alginate has been designated as Generally Recognized as Safe (GRAS) by the U.S. Food and Drug Administration (FDA) and is widely used as a pharmaceutical excipient.^18,19^ Recent studies have employed alginate-coated NPs to develop oral delivery-based carriers for administering various therapeutics. For instance, Nabipour et al. demonstrated that alginate coating on ZIF-8 NPs reduced burst release and enabled sustained release of curcumin at pH 5.8.^20^ A more recent strategy involves encapsulating NPs within polymeric microparticles (MPs), creating nano-in-micro drug delivery systems. These structures combine the advantages of both NPs and MPs, offering protection for encapsulated NPs and their payloads, minimizing burst release, and improving controlled drug release profiles.^21,22^ Dummert et al. recently achieved enhanced release kinetics for Thioflavin T by incorporating ZIF-8 NPs into alginate MPs.^23^ Table 1 summarizes recent advances on ZIF-8 NPs, alginate matrices, and their hybrid composite systems for controlled drug delivery, with a focus on their pH-responsive release profiles.

**Table 1:** Summary of Recent Advances in ZIF-8 and Alginate-Based Platforms for Controlled Drug Delivery

Authors	Year	Platform	Key Findings/Conclusion	Ref
Dummert et al.	2025	Thioflavin T-loaded ZIF-8/Alginate Matrix	• pH-responsive and tunable drug release behavior.	^23^
			• Reduced burst release to 12.5% h^–1^ within six hours.	
			• Nano-in-micro structures ensured consistent, controlled release.	
Nabipour et al.	2024	Curcumin-loaded ZIF-8/Alginate Coating	• High encapsulation efficiency (∼79%).	^20^
			• Alginate coating minimized burst release at acidic pH.	
			• Enhanced apoptosis in cancer cells.	
			• Confirming alginate’s stabilizing effect for MOF drug carriers.	
Frouhar et al.	2024	Alginate-Stabilized Curcumin–Selenium–ZIF-8 Nanocomposites	• Alginate enhanced colloidal stability and pH-responsive release.	^24^
			• Hemolytic activity significantly reduced (from ∼12.16% to 5.2%) after alginate coating.	
Mete et al.	2023	Sorafenib@ZIF-8 NPs	• High drug loading capacity with pH-responsive release.	^6^
			• Improved anticancer efficacy compared with free sorafenib.	
			• Suggested synergistic effect between sorafenib and released Zn^2+^ ions.	
Stalder et al.	2025	siRNA-Loaded LNPs in Alginate MPs	• Provided effective siRNA protection under gastric conditions.	^25^
			• pH-triggered release in intestinal environments.	
			• Preserved siRNA bioactivity after gastric exposure.	

Droplet microfluidics has emerged as an advanced technology for fabricating nano-in-micro delivery systems.^21,22^ This study suggests a novel, single-step method for fabricating pH-responsive nano-in-MPs via a water-in-oil microfluidics emulsion as an innovative platform for the oral delivery of sorafenib, which, to the best of our knowledge, has not been reported previously. Sorafenib was encapsulated within ZIF-8 NPs to ensure precise and sustained release. These drug-loaded NPs were then further encapsulated within alginate microparticles (aMPs). It was assumed that leveraging alginate’s pH-responsive properties would ensure the drug’s stability against the GI tract’s harsh environment and avoid premature release. It was also presumed that the design of nano-in-microparticles facilitated degradation of the aMP matrix upon passing the gut, resulting in swift distribution of the drug-loaded NPs in the intestine. ZIF-8 NPs exhibited a mean diameter of 72.18 nm with an EE of 76%. The resulting nano-in-microparticles indicated spherical morphology with hydrodynamic sizes ranging from 76 to 113 μm, depending on the flow rate ratio, and uniform dispersion of SZ NPs within the matrix. Drug release studies in simulated gastric and intestinal fluids revealed that the nano-in-microparticles prevented premature drug release in acidic simulated gastric fluid (pH < 5.7), while facilitating a controlled and sustained release profile under simulated intestinal conditions (pH 7.4) over 24 h. Importantly, the ZIF-8-based NPs continued to provide sustained drug delivery, maintaining therapeutic concentrations over an extended period via a controlled release mechanism. Cytotoxicity assays against HepG2 liver cancer cells revealed significantly enhanced anticancer efficacy for nano-in-microparticles compared to free sorafenib. These findings highlight the potential of this pH-responsive nano-in-microparticle platform as an effective oral delivery strategy for hepatocellular carcinoma therapy.

## 2 Materials and Methods

### 2.1 Materials

Alginate, acetic acid, dimethylsulfoxide (DMSO), zinc nitrate hexahydrate, 2-methylimidazole, span 80, and 3-(4,5-dimethylthiazol-2-yl)-2,5-diphenyl tetrazolium bromide (MTT) were obtained from Sigma-Aldrich (USA). Methanol (≥99.9%) was supplied by Merck. HepG2 cell line was received from the Pasteur Institute (Iran). Cell culture media consisting of Dulbecco’s modified Eagle’s medium (DMEM), fetal bovine serum (FBS), and penicillin-streptomycin was purchased from Gibco (USA). All chemicals and solvents were of analytical grade and utilized without further purification.

### 2.2 Synthesis of ZIF-8

ZIF-8 NPs were synthesized based on the previously established method of Wang et al. (2018),^26^ with slight modifications. Briefly, a solution was prepared by dissolving 2-methylimidazole (440 mg) and Zn (NO_3_)_2_·6H_2_O (200 mg) in 10 mL of methanol under ultrasonic conditions for 10 min, resulting in a clear solution for each component. Subsequently, the first solution was introduced into the second solution with stirring, with the resultant mixture being continuously stirred at 35 °C for 4 h to facilitate the formation of ZIF-8. The resultant product was isolated through centrifugation, subjected to three washes with methanol, and subsequently dried at 60 °C under vacuum overnight.

### 2.3 Preparation of Sorafenib-Loaded ZIF-8 (SZ NPs)

To synthesize SZ NPs, the same procedure outlined previously was employed. Specifically, 2.5 mg/mL sorafenib solution was prepared in methanol under ultrasonic conditions and blended with the dissolved 2-methylimidazole solution. Following a 5 min stirring period, this mixture was added to the dissolved zinc nitrate solution. The subsequent steps proceeded similarly to the ZIF-8 synthesis process. To better represent the encapsulation of ZIF-8 NPs into aMPs, ZIF-8 NPs were labeled with fluorescein isothiocyanate (FITC) and Rhodamine B (Figure S1, Supporting Information). This procedure was also carried out similarly to the sorafenib-loading into ZIF-8 NPs procedure (details in Supporting Information). The amount of untrapped sorafenib was determined based on the supernatant UV–vis absorption. The quantification of sorafenib encapsulated within ZIF-8 NPs was conducted referencing a calibration curve of sorafenib in methanol. The encapsulation efficiency was calculated as follows:

1 $$Encapsulation\;Efficiency\;\left(\%\right)=\frac{mass\;of\;drug\;in\;NP}{mass\;of\;total\;loaded\;drug}\times 100$$

### 2.4 Characteristics of ZIF-8 and SZ NPs

The morphologies and NPs dimensions were determined using field emission scanning electron microscopy (FE-SEM). Energy-dispersive X-ray (EDX) spectroscopy mapping was employed to determine elemental compositions. Crystalline phases were identified via X-ray diffraction (XRD) using a Philips X’Pert Pro diffractometer (Cu Kα radiation, λ = 1.541 Å, 40 kV, 25 mA). Fourier transform infrared (FT-IR) spectroscopy analysis was performed using a Bruker Equinox 55 spectrometer for determining the functional groups in both ZIF-8 and SZ NPs.

### 2.5 Microfluidic Device Fabrication and Assembly

Graphic of manuscript and Figure 9A present an overview of the microfluidics chip pattern. The master mold preparation proceeded according to the previously mentioned procedure.^27^ Briefly, the microchannel pattern was designed in SolidWorks 2016 (SolidWorks Corp). Subsequently, a silicon wafer was coated with SU2050 photoresist (Dow Corning) using a spin coater, ensuring a uniform height of 120 μm. Following a baking period for the SU-8, photolithography was performed using a chrome mask and UV exposure on a silicon wafer substrate. Unexposed SU-8 was removed through development with ethyl oxalate. Fabrication of the PDMS microfluidic chip proceeded by casting a 9:1 (w/w) mixture of PDMS base and curing agent (SILGARD 184, Dow Corning) onto the resulting master mold.

### 2.6 Numerical Study

To reduce computational complexity, the numerical Computational Fluid Dynamics (CFD) model and its experimental validation were performed using an alginate solution without ZIF-8 NPs. The flow-focusing junction was represented by a geometrical model discretized with a hexahedral nonuniform mesh, refined near the channel walls. Uniform inlet velocities were applied at both the dispersed and continuous phase inlets, while the channel walls were subjected to a no-slip boundary condition. A static contact angle of 160° was specified to represent the wetting behavior of the phases, and the outlet boundary was maintained at atmospheric pressure. The Pressure-Implicit with Splitting of Operators (PISO) algorithm was employed for pressure–velocity coupling, with spatial discretization achieved through a second-order upwind scheme. Pressure interpolation was performed using the Pressure Staggering Option (PRESTO) method, and temporal integration was carried out with a second-order implicit time-stepping scheme.

The model simulated the formation of non-cross-linked droplets by solving the incompressible Navier–Stokes equations for laminar flow, coupled with a two-phase Volume of Fluid (VOF) method to resolve the liquid–liquid interface. The model was used to investigate the influence of the flow rate ratio (Φ = Qd/Qc, where Qd and Qc denote the flow rates of the dispersed and continuous phases, respectively) on the size evolution of non-cross-linked droplets, cross-linked droplets, and purified MPs.

2 ∇·V=0

3 $$\frac{\partial \rho V}{\partial t}+\nabla \left(\rho V\cdot V\right)=-\nabla p+\nabla \cdot \mu \left(\nabla \cdot V+\nabla \cdot V^{T}\right)+F_{s}$$

4 $$F_{s}=\sigma \kappa \nabla \alpha$$

5 $$\frac{\partial \alpha }{\partial t}+\nabla \cdot \left(\alpha V\right)=0$$

where ***V*** and *p* denote the velocity and pressure fields, respectively. ***F***_*s*_ represents the continuum surface force, and α reflects the phase volume fraction within the computational cells. κ and σ represent the interface curvature and interfacial tension, respectively. The density (ρ) and dynamic viscosity (μ) of the mixture were determined using the α-weighted averages of the properties of the continuous (c) and dispersed (d) phases.

All of the aforementioned equations were solved using the finite element method.^28^ Figure 9A displays the flow-focusing device, which was recruited for numerical simulations. It is comprised of two inlets for injecting the continuous phase (mineral oil+3% span80), an inlet for injecting the dispersed phase (alginate solution), and an outlet at the downstream. The inlets and junction channels have a uniform width of 250 μm, with the outlet channel being 400 μm wide.^27^ Uniform velocity, atmospheric pressure, and no-slip boundary conditions were applied at inlets, outlets, and walls. The viscosity of dispersed and continuous phases was measured at a wide range of shear rates utilizing a Brookfield DV II Pro viscometer. The dispersed phase revealed shear-thinning and non-Newtonian behavior, and data were described, fitting to the cross model.^29^ The equation of the cross model and its parameters are outlined in Table 2. τ and *m* represent time constant with a time dimension and dimensionless rate constant, respectively. μ_0_, μ_∞_, γ̇, and μ also denote the zero-shear rate, infinite-shear rate viscosities, shear rate, and viscosity.

**Table 2:** Parameters of the Cross Model

Sample	Density	Viscosity (μ)	*m*	τ (s)	μ_0_	μ_∞_	Interfacial Tension
Dispersed phase	1002	$\frac{\mu -\mu_{\infty }}{\mu_{0}-\mu_{\infty }}=\frac{1}{1+{\left(\tau \mathrm{\gamma ̇}\right)}^{m}}$	0.445	0.0032	0.254	0	2.9
Continuous phase	840	0.038 pa·s			0.038	0.038	2.9

### 2.7 Fabrication of pH-Responsive SZ@aMPs (Droplet Formation, Gelation, and Size Control)

The microfluidic device employed in this study was composed of three consecutive units: a flow-focusing unit for droplet generation, an *in situ* gelling unit for pregelation, and a stabilizing unit for postprocessing (Graphic for manuscript and Figure 9A). Alginate aqueous solution 1 wt % (Sigma-Aldrich (A2033, medium viscosity)) (Mn = 208,000 g·mol-1, M/G = 1.2), prepared in calcium ethylenediaminetetraacetic acid (Ca-EDTA) solution, and introduced from inlet 3. In the flow-focusing unit, a mineral oil solution containing 3 wt.% Span 80 served as the continuous phase (inlet 2). Simultaneously, a mixture of mineral oil containing 0.2% v/v acetic acid and 3 wt.% Sapn80 was introduced through inlet 1 to reduce the pH and pregelation of sodium alginate droplets. After traversing a serpentine channel within the stabilizing unit, the pregelled microgels underwent final gelation in a 2 wt % CaCl_2_ bath. In order to load SZ into aMPs, 5 mg of SZ powder was weighed and added to the alginate solution, and then agitated for 2 h. The rest of the procedure was undertaken similarly to the free aMPs fabrication process.

### 2.8 Characterization of Initial Water-in-Oil Droplets, Solidified aMPs, and SZ@aMPs

The morphology and size distribution of water-in-oil droplets, cross-linked aMPs, and final purified MPs (without SZ NPs) were analyzed by an IX71 optical microscope (Olympus) equipped with a DP 6.3.0.5 online CCD camera. This procedure was also performed for the morphology characterization of SZ@aMPs with SEM microscopy and fluorescence microscopy. For determining the size distribution of the initial droplets and solidified MPs, the diameters of 100 droplets were measured to establish an average value. The coefficient of variation (CV), calculated as the standard deviation (SD) divided by the mean diameter and expressed as a percentage, was employed to characterize the size uniformity. For characterizing the morphology of SZ@aMPs and to confirm the successful loading of fluorescently labeled SZ NPs into aMPs, SEM and fluorescence microscopy analysis were conducted.

### 2.9 *In Vitro* Drug-Release Studies

The *in vitro* release kinetics of sorafenib from freeze-dried SZ NPs loaded nano-in-microparticles were ascertained under conditions simulating the distinct pH environments of the gastrointestinal tract. Release studies were carried out in phosphate-buffered saline (PBS) at pH 1.2 (simulating gastric conditions), 5.7 (intestinal conditions), and 7.4 (colonic conditions) at 37 °C and continuous shaking at 100 rpm. Specifically, 1 mg of SZ@aMPs was dispersed in 1 mL of PBS solution within dialysis bags (MWCO = 3.5 kDa) and immersed in 30 mL of the respective PBS solution. At predetermined time intervals, samples of the dialysis exudate were withdrawn and replaced with an equal volume of fresh PBS solution to maintain sink conditions. UV absorbance analyses were undertaken to evaluate the release profiles of the collected samples. The percentage of sorafenib release from the SZ@aMPs was calculated using the following formula:

6 $$Sorafenib\;release\;\left(\%\right)=\frac{free\;drug}{total\;drug\;loaded}\times 100$$

### 2.10 Cell Culture and *In Vitro* Cytotoxicity Study

Human hepatocellular carcinoma (HepG2) cells were cultured in DMEM containing 10% FBS and 1% penicillin/streptomycin and incubated at 37 °C under standard conditions (5% CO_2_). HepG2 cells were seeded in 96-well plates at 10^4^ cells/well for cytotoxicity assays and incubated for 24 h to enable cell attachment. The medium was then replaced with varying concentrations (6.25, 12.5, 25, 50, and 100 μg/mL) of sorafenib and SZ NPs. Cell viability was ascertained using the MTT assay (5 mg/mL MTT, 4 h, 37 °C), followed by DMSO solubilization and absorbance measurement at 570 nm.

### 2.11 Apoptosis Analysis

To explore the cytotoxic effects of SZ NPs on HepG2 cells, an apoptosis assay by Annexin V-FITC/propidium iodide (PI) was undertaken using the flow cytometry analysis method. The cells were seeded in 6-well plates at a density of 5 × 10^5^ cells/mL and incubated at 37 °C under standard cell culture conditions. After 24 h, a fresh medium containing varying amounts of SZNPs (2 mL/well) was replaced with the culture medium. Following 24 h of incubation under the same conditions, the cells were harvested using trypsinization and centrifuged at 800 rpm for 5 min. Subsequently, the cell pellet was resuspended in 250 μL of binding buffer. Next, 2 μL of Annexin V-FITC and 5 μL PI were added, with the mixture being incubated for 15 min at room temperature; the stained cells were analyzed for apoptotic effects induced by the test compounds using a flow cytometer.

### 2.12 Statistical Analysis

All experiments were conducted in triplicate to ensure reliability and consistency. Data were analyzed by presenting the findings as mean values ± SD. One-way analysis of variance (ANOVA) was employed to assess statistical significance (*p < 0.05, **p < 0.01, ***p < 0.001).

## 3 Results and Discussion

### 3.1 Mathematical Modeling

To better predict the behavior of alginate solution in microfluidics channels and the effects of variation in parameters such as capillary number (Ca) and flow rate ratios (Φ), on the size of droplets, a CFD study was applied before initiating the experimental section of the project. The experimental and numerical values of the droplet diameter with their relative errors are reported in Table 3. It is observed that the size of droplets of 1% Na-alginate solution is in excellent agreement with experimental data under different physical conditions.

**Table 3:** Experimental and Numerical Droplet Diameters

Φ	Ca	*D* (solidified washed)	*D* (solidified unwashed)	*D*_exp_ of initial droplets	*D*_sim_ of inititial droplets	Error %
0.02	Ca = 0.024	76	130	168	185	10
0.04	Ca = 0.012	113	180	217	244	1
0.08	Ca = 0.012	134	205	252	255	12

Figure 1 illustrates a comparison of the results of numerical CFD and experimental procedure images of droplet formation gained by bright field microscopy under different flow conditions: Ca = 0.024 and Φ = 0.02 (Figure 1A), Ca = 0.012 and Φ = 0.04 (Figure 1B), and Ca = 0.012 and Φ = 0.08 (Figure 1C). These numerical schematics are compared with the corresponding experimental observations shown in Figures 2–4, respectively. The numerical results indicate that upon elevating the (Φ = Qd/Qc) from 0.02 to 0.08, the size of yielded droplets and the frequency of the droplet formation increased. On the other hand, upon augmenting the capillary number from 0.012 to 0.024, the droplet diameter diminished significantly. The MPs’ size was ascertained at three distinct stages: immediately postfabrication droplets, solidified but unwashed, and solidified and washed MPs (Figures 2–4). Microscopy images illustrating the morphological evolution and size distribution at each stage are presented for different flow conditions in Figure 2 (Φ = 0.02), Figure 3 (Φ = 0.04), and Figure 4 (Φ = 0.08). Each figure pairs microscopy images with their corresponding size distribution for the initial droplets (panels (A, B)), unwashed (panels (C, D)), and washedstates (panels (E, F)). This suggests that as FRRs increased, so did the microparticle sizes (Table 3).

**Figure 1:**
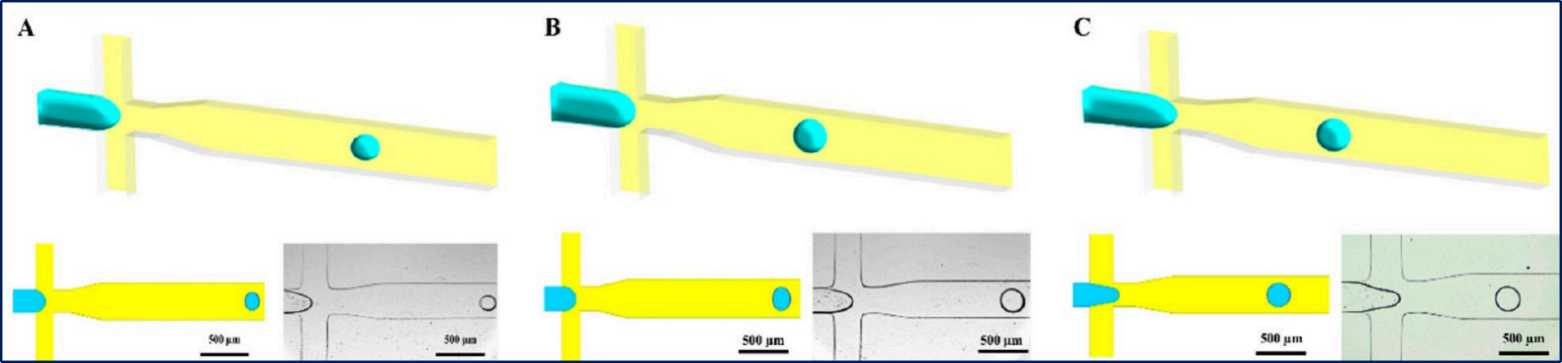
Results of a numerical study under varying capillary numbers (Ca) and flow rate ratios (Φ). Numerical study for (A) Ca = 0.024 and Φ = 0.02; (B) Ca = 0.012 and Φ = 0.04; (C) Ca = 0.012 and Φ = 0.08.

**Figure 2:**
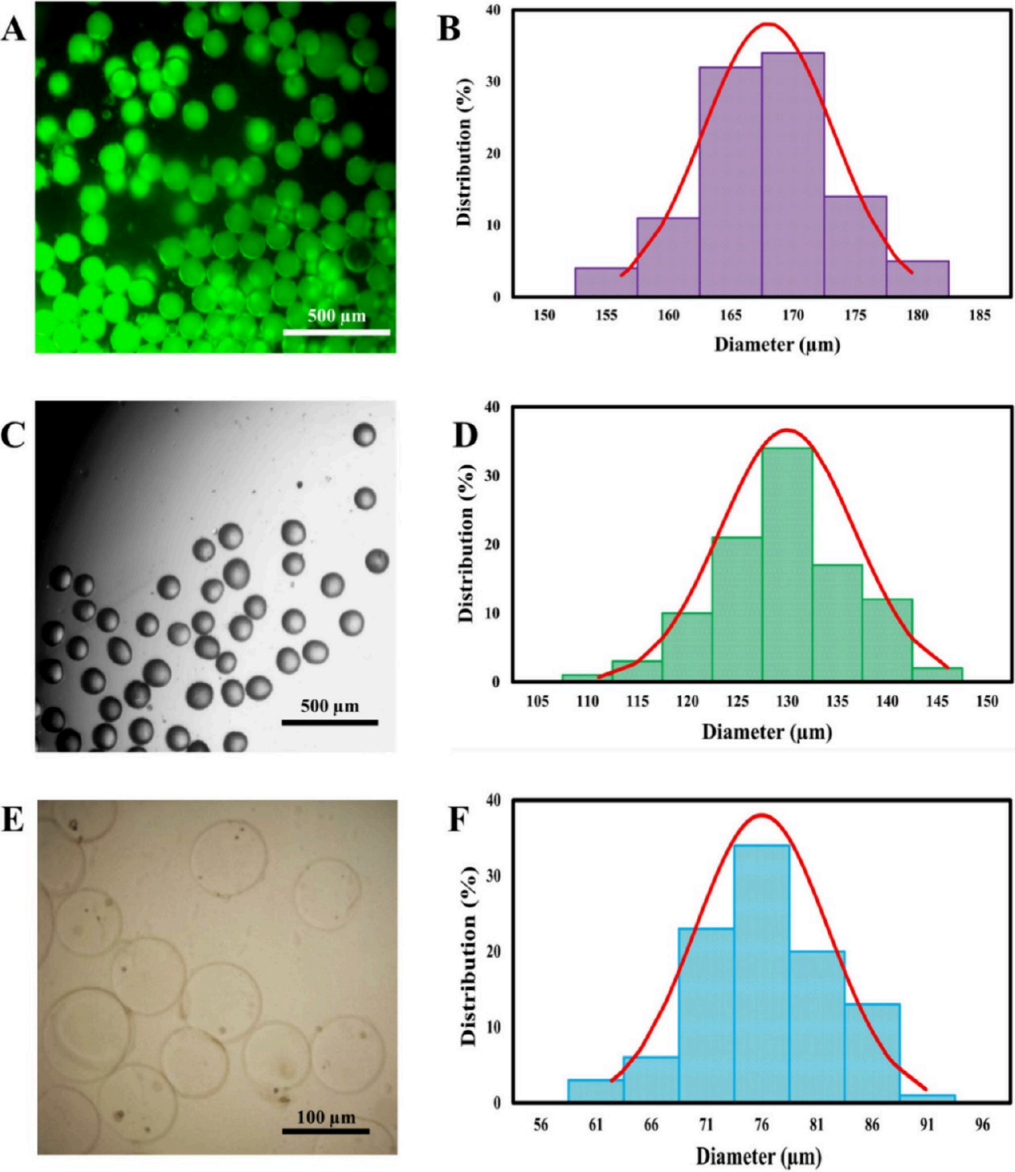
Characterization of droplet morphology and size distribution. Representative microscopy images and corresponding size distributions for droplets (Ca = 0.024, Φ = 0.02) at different processing stages: (A, B) initial droplets, (C, D) unwashed, and (E, F) washed.

**Figure 3:**
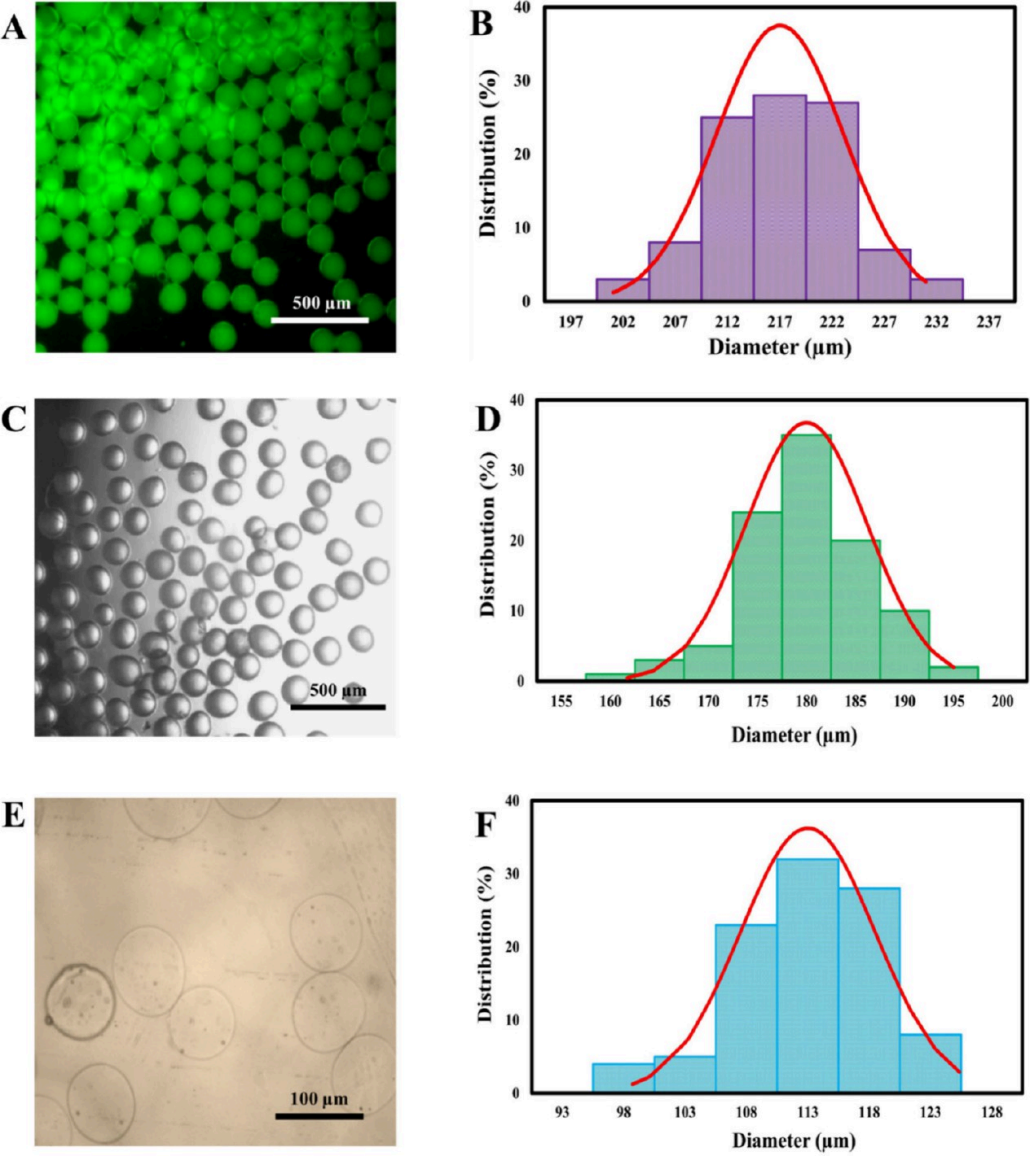
Characterization of droplet morphology and size distribution. Representative microscopy images and corresponding size distributions for droplets (Ca = 0.012 and Φ= 0.04) at different processing stages: (A, B) initial droplets, (C, D) unwashed, and (E, F) washed.

**Figure 4:**
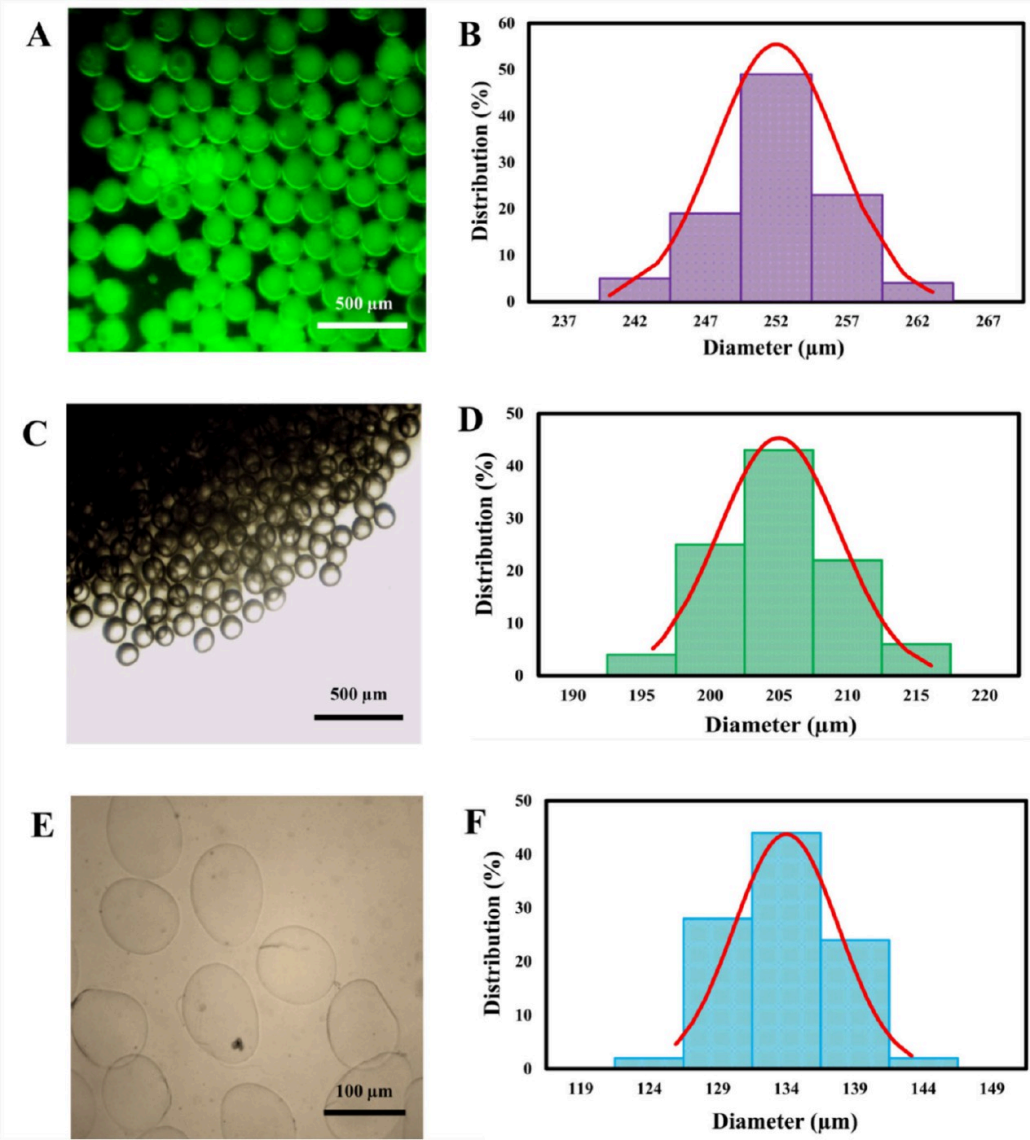
Characterization of droplet morphology and size distribution. Representative microscopy images and corresponding size distributions for droplets (Ca = 0.012 and Φ= 0.08) at different processing stages: (A, B) initial droplets, (C, D) unwashed, and (E, F) washed.

Based on the results, at a constant alginate concentration, at low capillary numbers, and flow rate ratios, the most dominant regimes of droplet formation were dripping and binary dripping. On the other hand, elevation of the capillary number or flow rate ratios led to more unstable droplet formation regimes, with a transformation of the dripping regime toward binary dripping and quasi-jetting regimes.^27^ The results of our study also concurred with other studies exploring the droplet formation regimes in microfluidic channels.^30–32^ The results were in accordance with our previous research in which we employed a numerical CFD to examine various droplet formation regimes using a device with the same rheological characteristics, channel width, and alginate concentration.^27^

### 3.2 ZIF-8 NP and SZ NP Characterization

ZIF-8 was synthesized using Zn^2+^ ions and 2-methylimidazole as precursors. This method can yield ZIF-8 reproducibly with the desired sodalite topology. The utilization of methanol as a solvent can promote rapid ligand deprotonation and coordination with Zn^2+^, facilitating the formation of uniformly sized nanocrystals under mild conditions (35 °C), an essential characteristic for efficient cellular uptake and drug delivery applications.^4,33^ Further, a controlled excess of 2-methylimidazole may enhance this process by accelerating ligand coordination, stabilizing nascent nanocrystals, and suppressing Ostwald ripening, thereby offering a precise control over particle size distribution.^34^ Compared with conventional solvothermal or hydrothermal methods, this low-temperature method can eliminate the need for harsh reagents, elevated temperatures, and high-pressure autoclaves, factors that could otherwise compromise drug stability during encapsulation. In addition, its simplicity and scalability could also eliminate the need for energy-intensive equipment, making the process cost-effective and suitable for large-scale production.^35^

SZ NPs were also fabricated using a facile, one-pot coprecipitation and encapsulation strategy. This method benefits from the inherent interactions between sorafenib and the ZIF-8 precursors. Initially, a solution of sorafenib and 2-methylimidazole was prepared. The aromatic rings present in both sorafenib and 2-methylimidazole may facilitate π–π stacking interactions, promoting the preorganization of sorafenib molecules within the imidazole linker network. Further stabilization can be obtained through hydrogen bonding between sorafenib’s amide/urea groups and the imidazole NH groups. These noncovalent interactions (π–π stacking, hydrogen bonding, van der Waals forces, and hydrophobic packing) may boost sorafenib’s solubility and promote its efficient encapsulation within the growing ZIF-8 framework.

Subsequent addition of this mixture to a zinc nitrate solution would trigger rapid ZIF-8 self-assembly. Zn^2+^ ions coordinate with deprotonated imidazolate to form the tetrahedral Zn–N framework. Concurrently, sorafenib molecules become physically confined within the forming pores (11.6 Å cages, 3.4 Å apertures) and defect sites, primarily through physical entrapment and supramolecular interactions rather than covalent bonding. Owing to its relatively large molecular size, sorafenib is predominantly encapsulated along crystal growth rather than via postsynthesis diffusion. This *in situ* encapsulation approach yields high encapsulation efficiencies, surpassing those typically achieved using postsynthesis methods.^6,12,36^

The synthesized NPs exhibited a characteristic hexagonal morphology, as visualized by FE-SEM imaging (Figure 5A, B). ImageJ analysis of the NPs revealed a size distribution ranging from 45 to 130 nm, with an average diameter of 72.18 ± 1.1 nm (Figure 5C). Furthermore, EDX analysis confirmed the successful formation of ZIF-8, evidenced by the distinct presence of C, N, O, and Zn (Figure 6A-C).

**Figure 5:**
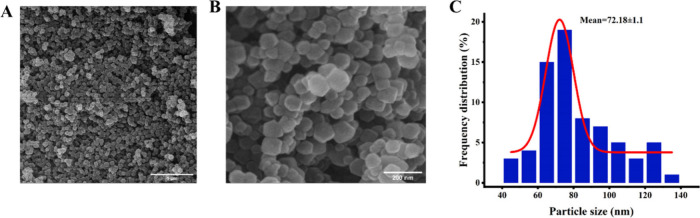
Morphological and size characterization of ZIF-8 NPs. (A, B) Representative FE-SEM images at low and high magnification (scale bars: 1 μm and 200 nm, respectively). (C) Size distribution profile.

**Figure 6:**
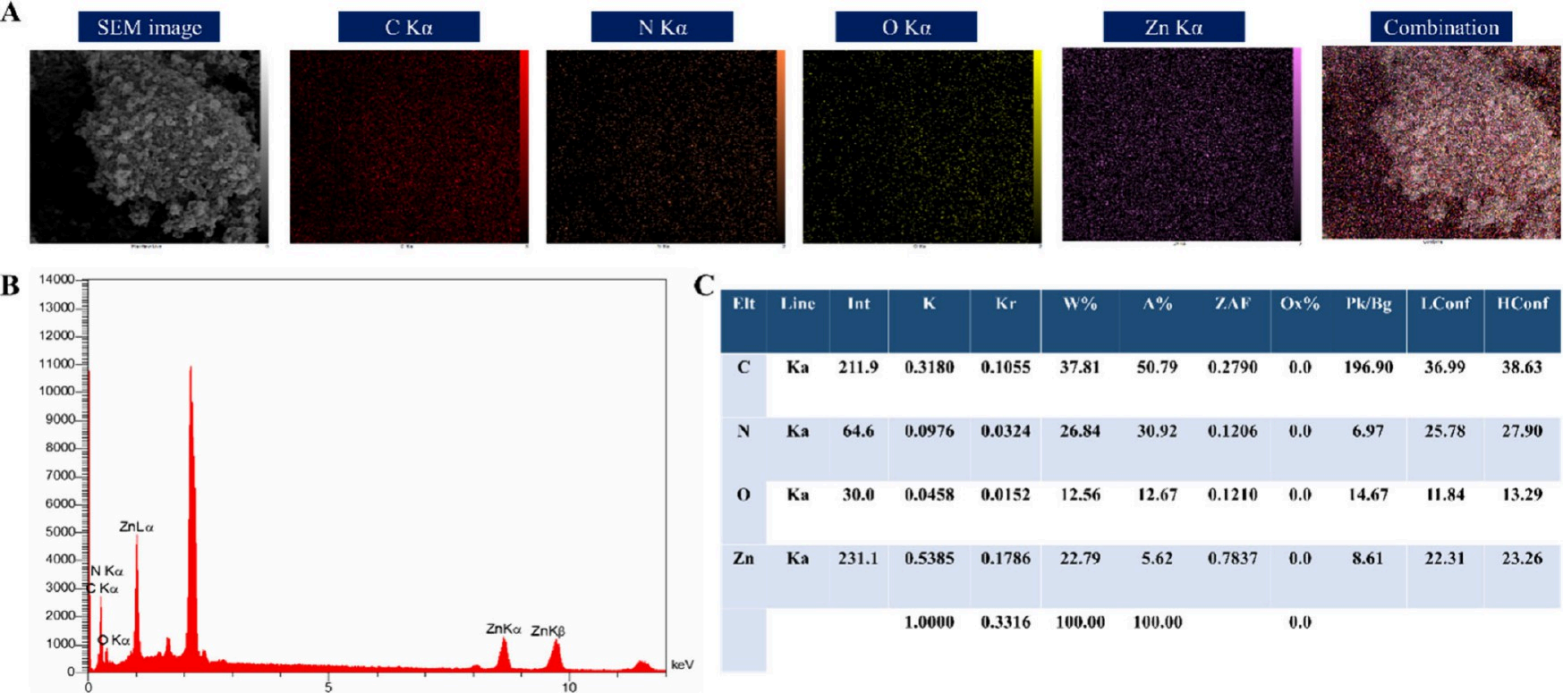
Elemental analysis of ZIF-8 NPs. (A) EDX elemental mapping. (B) Representative EDX spectrum. (C) Quantitative elemental compositions.

SZ NPs were synthesized using a one-pot approach. Sorafenib was premixed with the ligand solution, ensuring uniform distribution and facilitating incorporation during ZIF-8 crystallization. Importantly, sorafenib may not solely be trapped within preformed pores but may also act as an auxiliary ligand during crystallization. The nitrogen atoms present in its urea and pyridine-like functional groups can potentially compete with the nitrogen atoms of 2-methylimidazole for Zn^2+^ coordination, allowing sorafenib to integrate into the crystal lattice itself.^37,38^ This could contribute to high loading efficiency and enhanced drug retention.

As the nanocrystals mature, additional noncovalent interactions may further stabilize sorafenib within the framework. Hydrophobic partitioning may drive localization of the drug into the apolar interior of the 2-methylimidazolate-lined pores, lowering solvent exposure. Further, π–π stacking interactions between sorafenib’s aromatic moieties and the imidazolate linkers, along with hydrogen bonding, may strengthen drug affinity and stability within the framework.^39–41^ These interactions underpin the high encapsulation efficiency. The encapsulation efficiency (EE) of sorafenib within ZIF-8 NPs was specified using UV–vis spectroscopy and calculated using Equation 1, yielding a value of approximately 76%. This can be attributed to both synthesis optimization (e.g., drug-to-precursor ratio, the timing of drug introduction) and innate physicochemical affinities. Specifically, the hydrophobic and aromatic drug molecules present strong compatibility with the porous, imidazolate-based ZIF-8 structure, facilitating efficient incorporation and retention during crystallization. Thus, this high loading capacity is crucial for developing effective nanoformulations designed to overcome the bioavailability challenges of free sorafenib. Notably, the EE achieved in this study is in line with or exceeds values reported in previous studies,^1,6,42^ highlighting the effectiveness of our synthesis approach in generating well-loaded NPs suitable for oral drug delivery.

The phase structure and crystallinity of ZIF-8, sorafenib, and SZ were analyzed using X-ray diffraction (XRD) (Figure 7, 5° ≤ 2θ ≤ 80°). The XRD pattern of ZIF-8 presented characteristic diffraction peaks at 2θ values of 7.4°, 10.2°, 12.6°, 14.8°, 16.4°, 18.2°, 19.4°, 22.1°, 24.3°, 26.8°, and 29.8°, corresponding to the (011), (002), (112), (022), (013), (222), (114), (233), (134), and (044) crystallographic planes respectively, consistent with literature reports, confirming its high crystallinity.^43^ Sorafenib indicated distinct diffraction peaks at 2θ = 13.2°, 17.8°, and 21.5°, also suggesting its crystalline structure. In the SZ NPs pattern, the primary diffraction peaks of ZIF-8 were preserved, confirming that the crystalline integrity and overall lattice structure of ZIF-8 were retained following the incorporation of sorafenib. Comparison with the pure sorafenib XRD pattern revealed the absence of characteristic sorafenib peaks. This could suggest that sorafenib is not present as a separate crystalline phase on the surface of ZIF-8; otherwise, distinct peaks attributable to sorafenib would have appeared superimposed on the ZIF-8 pattern. The lack of crystalline sorafenib peaks in the SZ pattern might provide evidence that the drug molecules were successfully encapsulated within the ZIF-8 pores. Within the framework, sorafenib may exist in a molecularly dispersed form, could lose its long-range crystalline order, and thus its distinctive diffraction signature. Nevertheless, subtle modifications were observed in the SZ pattern, including slight peak shifts toward lower angles and reductions in peak width, suggesting minor lattice expansion and enhanced structural ordering. These changes may suggest minor lattice expansion and improved structural ordering, in accordance with guest molecules applying internal pressure on the framework. These variations can be attributed to host–guest interactions between sorafenib molecules and the ZIF-8 framework, including hydrogen bonding and π–π interactions, which stabilize the composite structure and restrict molecular mobility. These interactions could stabilize the composite structure, restrict molecular mobility, and facilitate the successful encapsulation of sorafenib within the ZIF-8 pores rather than superficial adsorption.

**Figure 7:**
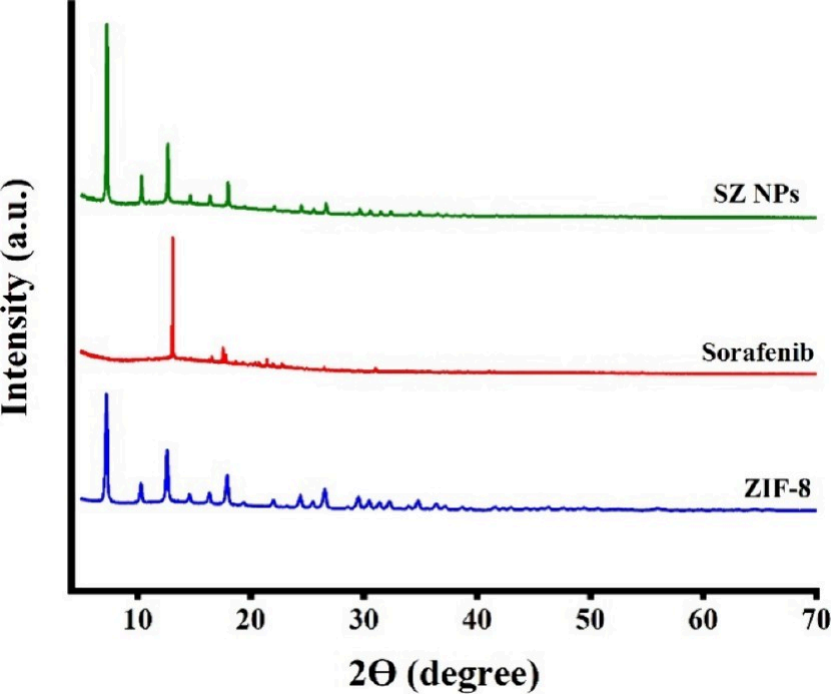
X-ray diffraction (XRD) analysis. Patterns for pristine ZIF-8, free sorafenib, and synthesized SZ NPs.

Fourier-transform infrared (FTIR) spectroscopy (Figure 8) confirmed the presence of characteristic vibrational bands corresponding to sorafenib, ZIF-8, and SZ, thereby revealing the molecular interactions involved in drug encapsulation. The FTIR spectrum of sorafenib presented the characteristic N–H stretching vibration at 3209 cm^–1^, a carbonyl (C=O) band at 1722 cm^–1^, and amide C=O and N–H bending vibrations at 1687 and 1600 cm^–1^, respectively. Additional peaks included C–N stretching at 1235 cm^–1^, C–F/C–Cl vibrations of the chlorotrifluoromethyl group at 1314 cm^–1^, along with aromatic out-of-plane C–H bending at 838 cm^–1^ and N–H wagging at 678 cm^–1^, in accordance with the literature.^6^ The ZIF-8 spectrum illustrated characteristic imidazolate framework vibrations, including aromatic C–H stretching in the 3200–3000 cm^–1^ region, aliphatic C–H stretching at 2928 cm^–1^, and imidazolate-related C–N and ring vibrations at 1584, 1433, and 1310 cm^–1^. Peaks within 1350–900 cm^–1^ were assigned to in-plane bending of the imidazolate linker, while absorptions at 757 and 690 cm^–1^ corresponded to out-of-plane sp^2^ C–H bending, verifying the structural integrity of the ZIF-8 framework.

**Figure 8:**
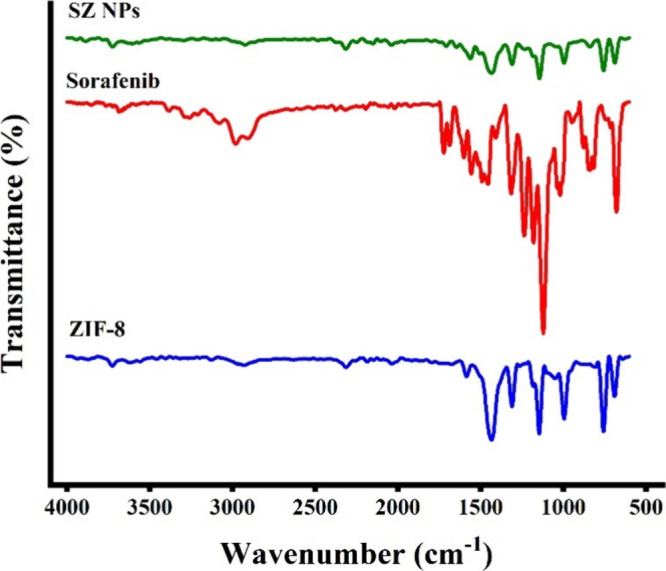
Fourier-transform infrared (FTIR) spectroscopy analysis. Spectra of ZIF-8, sorafenib, and SZ NPs.

The FTIR spectrum of SZ retained the characteristic ZIF-8 bands, reflecting preservation of the framework upon drug incorporation, whereas notable spectral changes revealed interactions between sorafenib and the carrier. Specifically, the sorafenib C=O stretch shifted from 1722 to 1707 cm^–1^, which may be assigned to hydrogen bonding between the carbonyl group and imidazolate nitrogen. Broadening and attenuation of the 3200–3000 cm^–1^ envelope may suggest involvement of the N–H groups in hydrogen bonding with ZIF-8. Further, the enhanced intensity and sharpening of the linker vibrations at 1561 and 1432 cm^–1^ might indicate perturbations of the imidazolate environment upon drug incorporation. The retention of sorafenib’s aromatic band at 838 cm^–1^ confirmed successful encapsulation, while the sharpening of the 688–690 cm^–1^ band could reflect π–π or CH−π interactions between sorafenib aromatic rings and the imidazolate linker, as well as confinement within the ZIF-8 pores. Notably, these spectral modifications not only may confirm molecular-level interactions but also may reflect the in situ encapsulation process, whereby sorafenib molecules participate in the nucleation and growth of ZIF-8 crystals. The observed C=O downshift and N–H perturbations and modulation of imidazolate vibrations might suggest that sorafenib stabilizes within the developing framework through hydrogen bonding, whereas π–π and CH−π interactions further anchor the drug within the porous matrix. In addition, the absence of major disruptions to ZIF-8’s fingerprint vibrations could also confirm that the carrier preserves its structural integrity, while the incorporation of sorafenib along crystal formation ensures its uniform distribution inside the framework rather than superficial adsorption.’

### 3.3 Characterization of SZ@aMPs

A microfluidic-based strategy was developed to fabricate uniform, spherical SZ@aMPs, adapting a previously reported hybrid on-chip/off-chip gelation technique.^44^ As illustrated in Figure 9A, the microfluidic device consisted of three sequential modules: droplet generation, on-chip semisolidification, and off-chip stabilization. The process initiated with the formation of monodisperse water-in-oil droplets at the flow-focusing junction. These droplets then underwent an initial acid-induced pregelation within the solidification segment before being collected in a CaCl_2_ solution for complete solidification. The size evolution of the droplets during fabrication is depicted in Figure 9B. The solidified, unwashed MPs exhibited diameters of approximately 130, 180, and 205 μm at Φ of 0.02, 0.04, and 0.08, respectively (Figures 3A, 4A, 5A). Following purification, the hydrodynamic diameters decreased to 76, 113, and 134 μm (Figures 3C, 4C, and 5C), reflecting matrix compaction and solvent removal during cross-linking. The successful generation of solid, spherical aMPs loaded with SZ was confirmed by bright-field microscopy, with representative images provided in the Figure S1 (Supporting Information).

**Figure 9:**
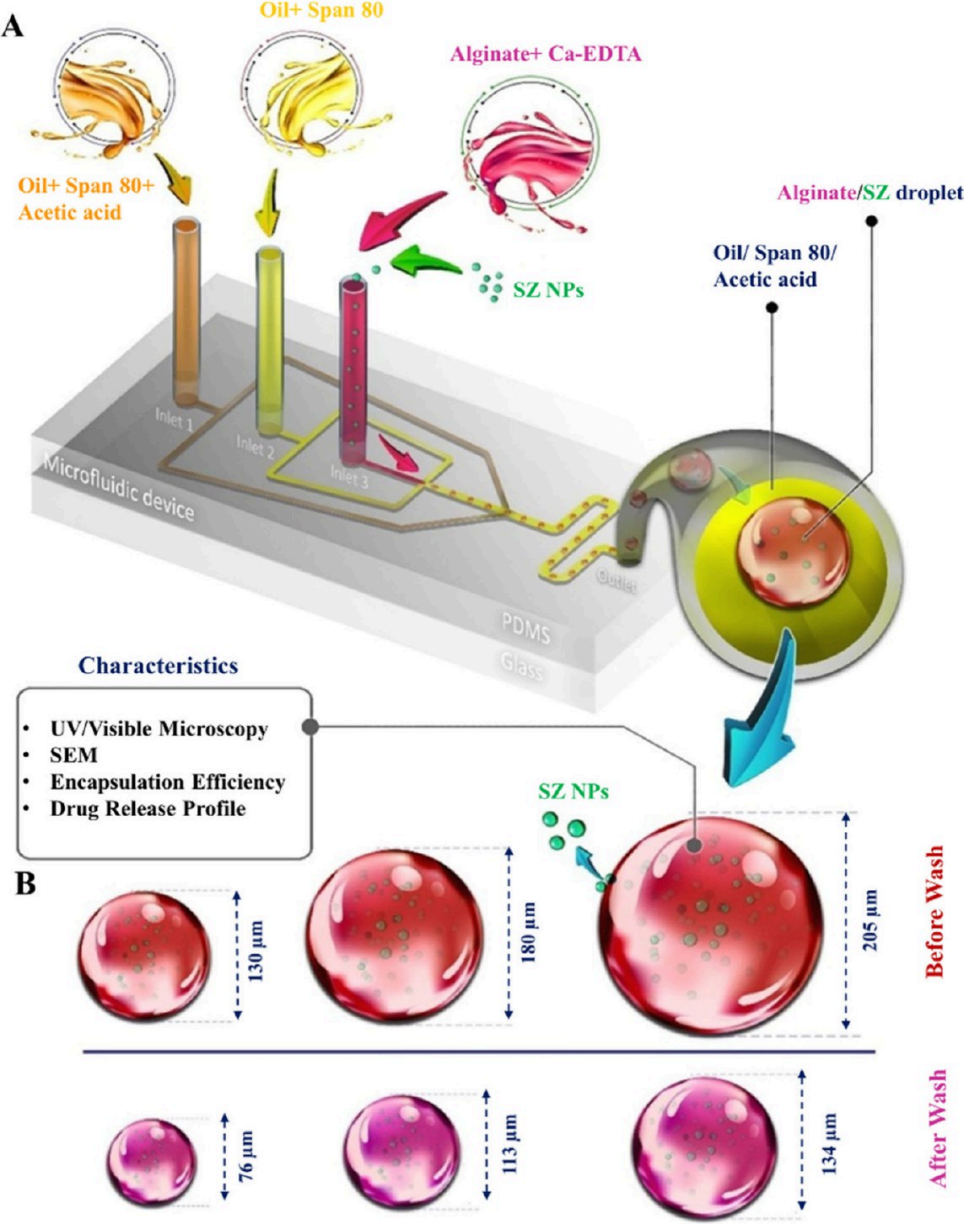
(A) Schematic representation of the microfluidic fabrication process for tunable SZ@aMPs. (B) Schematic illustration of microparticle size before and after washing.

The morphological and structural characteristics of the synthesized SZ@aMPs were analyzed using FE-SEM and optical/fluorescence microscopy. FE-SEM analysis (Figure 10) confirmed the spherical geometry of the aMPs and, at higher magnification, revealed the distinctive hexagonal morphology of the encapsulated ZIF-8 NPs, consistent with the morphology of pristine ZIF-8 shown in Figures 5A, B. The successful encapsulation and homogeneous distribution of the NPs within the alginate matrix were further verified by fluorescence microscopy. As shown in the paired brightfield and fluorescent images (Figure 11A, B), the fluorescently labeled ZIF-8 NPs (green, FITC channel) were homogeneously distributed throughout the aMPs. This was corroborated by optical/fluorescence images using rhodamine B (Figure S2, Supporting Information). These collective results demonstrate that the alginate network forms a tightly cross-linked, pH-responsive matrix that effectively entraps and uniformly distributes the ZIF-8 NPs, thereby ensuring the structural integrity of the encapsulated phase.

**Figure 10:**
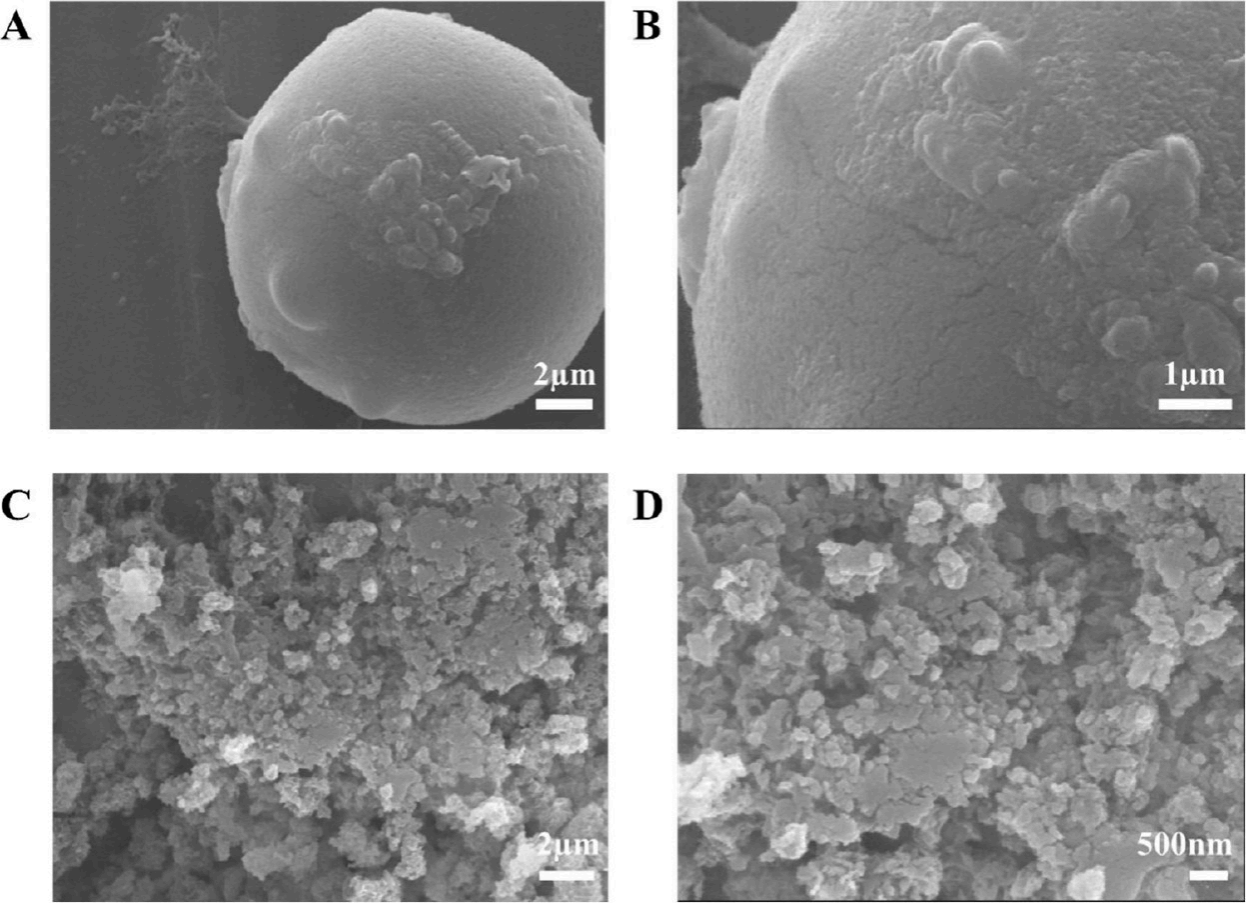
Morphological characterization of SZ@aMPs. (A, B) FE-SEM images showing the overall spherical morphology of the SZ@aMPs (Scale bars: 2 and 1 μm, respectively). (C, D) FE-SEM images of hte SZ@aMP surface (Scale bars: 2 μm and 500 nm, respectively).

**Figure 11:**
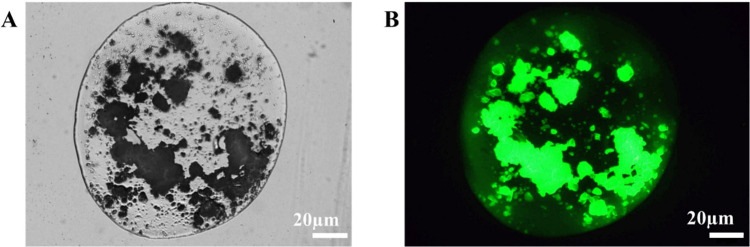
Optical and fluorescent microscopy of SZ@aMPs. (A) Brightfield microscopy image of SZ@aMPs (Scale bar: 20 μm). (B) Fluorescent image of the same field of view labeled by FITC (Scale bar: 20 μm).

The significance of these findings is underscored when contrasting the microfluidic approach with conventional encapsulation strategies, as detailed in Table 1. While methods such as ionic gelation and extrusion dripping are simple and scalable, they often yield particles with broad size distributions with limited morphological control. This heterogeneity can lead to unpredictable drug release profiles and bioavailability.^45,46^ Although electrospraying improves particle uniformity, its sensitivity to solution properties and electrical parameters can pose challenges for robust, reproducible formulation.^47,48^ In contrast, the microfluidic approach enables highly controlled droplet generation with exceptional reproducibility and structural integrity. The confined microchannel environment allows for precise control over droplet size, residence time, and interfacial dynamics, ensuring consistent gelation and minimizing NPs aggregation or leakage.^49,50^ Moreover, the microfluidic technique offers the unique capability to produce highly monodisperse particulate structures with near-complete encapsulation efficiency,^51^ while allowing simultaneous control of multiple fluid streams to assemble complex nano-in-micro architectures in a single step.^29,52^

### 3.4 *In Vitro* Drug-Release Studies

In order to obtain an oral drug delivery system and ensure controlled drug release in the gastrointestinal tract, drug-loaded microcapsules need to possess pH-responsive characteristics. Varying PBS solutions were utilized to represent the pH of different gastrointestinal tract regions: pH 1.2 simulated gastric juice, pH 5.7 represented intestinal fluid, and pH 7.4 simulated blood circulation.

The drug release profile of SZ@aMPs was highly pH-dependent, as shown in Figure 12. At pH 1.2, the aMPs remained intact over 48 h, effectively protecting the encapsulated SZ NPs from degradation under harsh physiological conditions of the stomach. Alginate-based hydrogels present a strong sensitivity to pH, a property that plays a key role in their swelling behavior, degradation rate, and controlled drug release. The sensitivity of alginate to pH changes is attributed to the presence of carboxyl groups (−COOH) within its molecular structure. Under acidic conditions, where pH is lower than its p*K*_a_ (pH < 3.4), these carboxyl groups remain protonated, causing alginate to stay unattacked. This stable protonated form results in minimal release of SZ NPs from the alginate matrix. This characteristic is particularly beneficial for oral drug delivery applications.^53^ The stability of ZIF-8 NPs is highly pH-dependent. Within the ZIF-8 structure, 2-methylimidazolate linkers are responsible for the sodalite-type porous structure of NPs, with tetrahedrally coordinated as linkers to zinc ions. In the absence of protective alginate matrices, ZIF-8 NPs would normally experience rapid degradation in the stomach owing to complete linker protonation. The fabrication of nano-in-micro structures has recently shown promise, facilitating the oral delivery of fragile therapeutics such as antibodies, proteins, and ribonucleotides.^54–56^ Previously, Stalder et al. employed alginate as gastro-resistant MPs to preserve siRNA-loaded lipid NPs from the harsh environment of the gut to develop an oral delivery system for TNF-á siRNAs.^55^

**Figure 12:**
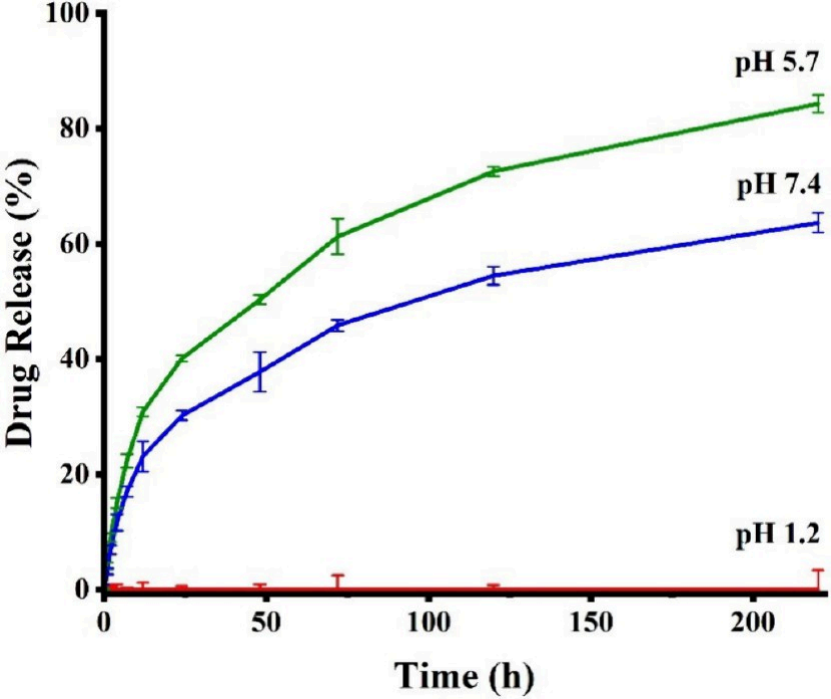
pH-dependent drug release profile from SZ@aMPs. Cumulative sorafenib release from SZ@aMPs (pH 1.2, 5.7, and 7.4).

As the pH approaches 5.7, which remains above the p*K*_a_ of the carboxyl group (∼3.4), the carboxyl groups stay in their deprotonated state, thereby enhancing the swelling and solubilization of the aMPs.^57^ On the other hand, in a physiological medium where monovalent cations (Na^+^, K^+^) are present, divalent Ca^2+^, acting as cross-linkers in solid microparticle structures, are gradually replaced. This ionic exchange also extends the alginate matrix degradation. The higher the pH is, the greater the degradation rate of the alginate matrices. Above pH = 5, the degradation of the alginate accelerates, leading to sustained release of SZ NPs from the aMPs. Over the first 4 h in the simulated intestinal fluid (pH 5.7), approximately 15% of sorafenib was released from the hydrogel MPs. The cumulative amounts of sorafenib released reached 30% and 40% during 12 and 24 h, respectively, and ultimately reached 84.2% during 220 h, indicating successful sustained release. The obtained results were in accordance with past research.^12^ Once SZ NPs are released into the weak acidity of the pH = 5.7, these NPs could change as a pH-responsive drug release; the porous structure of ZIF-8 exhibits degradation within acidic conditions,^4,58^ facilitating the controlled release of the encapsulated drug. pH-sensitive drug delivery systems improve therapeutic efficacy by concentrating drug release within the tumor and reducing off-target effects.^59^ pH-sensitive drug delivery system improves therapeutic efficacy by concentrating drug release within the tumor and reducing off-target effects.^59^

At pH 7.4, the physiological deprotonation of alginate carboxyl groups induces swelling and progressive disintegration of the MPs matrices. This facilitates the efficient release of encapsulated SZ NPs, enabling ZIF-8 NPs to be liberated into the intestinal environment, penetrate mucosal barriers, and ultimately enter systemic circulation. If the sorafenib was directly loaded in the aMPs, most of the drug would be rapidly released within a short time, and cleared from the intestine quickly. Nevertheless, at this pH, the ZIF-8 framework is the most stable, leading to the slowest drug release. The metal–ligand coordination bonds remained largely unaffected, and the dense structure of ZIF-8 retained its integrity, ensuring a controlled and sustained drug release. The total sorafenib released from the hydrogel microparticle along the first 4 h at pH = 7.4 was about 11%. The cumulative amounts of sorafenib released reached about 23% and 30% after 12 and 24 h, respectively, and finally 63.6% during the 220 h. These findings suggest that sorafenib release was slow and sustained over time. This pattern of drug release at pH = 7.4 has been confirmed by Dummert et al., where encapsulation of ZIF-8 NPs into alginate nanoparticles led to slower release of Thioflavin T (12.5% in 6 h).^23^

### 3.5 *In Vitro* Cytocompatibility

Cell viability in the HepG2 cells was assessed following exposure to varying concentrations (6.25–100 μg/mL) of free sorafenib and SZ NPs after 48 h using the MTT assay (Figure 13). Both free sorafenib and SZ NPs presented concentration-dependent cytotoxicity, with cell viability diminishing as concentration increased. At 6.25 μg/mL, free sorafenib lowered viability to approximately 86%, while at 100 μg/mL, viability diminished to approximately 50%. SZ NPs revealed similar trends, with 74% and 40% viabilities at 6.25 and 100 μg/mL, respectively. SZ NPs consistently indicated lower cell viability than free sorafenib at all concentrations. This enhanced cytotoxicity is particularly pronounced at higher concentrations (50 and 100 μg/mL). When alginate matrices start to degrade, SZ NPs are distributed in the cell culture medium. In this pH, SZ NPs remain intact, preventing the release of sorafenib, its metabolism, and chemical modifications. In contrast, for the free sorafenib added to the cell culture, metabolism and structural modification take place faster. Also, SZ NPs can be taken up more effectively by HepG2 cells and release sorafenib in the acidic pH of the lysosome, leading to more cytotoxic effects for this type of sorafenib compared to free sorafenib. This pattern of cell cytotocicity was also confirmed by previous publications.^6,20^ Previously, Li et al. have also reached the same cytotoxicity effects for SZ NPs applied on the Huh-7 cell line.^7^ Nabipour et al. used a layer of alginate coating on the curcumin-loaded ZIF-8 to diminish the curcumin burst release, while having a sustained release behavior. The results of the MTT assay on HeLa, HEK293, and SH-SY5Y cells revealed a remarkable cytotoxicity for CUR@Zn-MOF and SA/CUR@Zn-MOF.^20^

**Figure 13:**
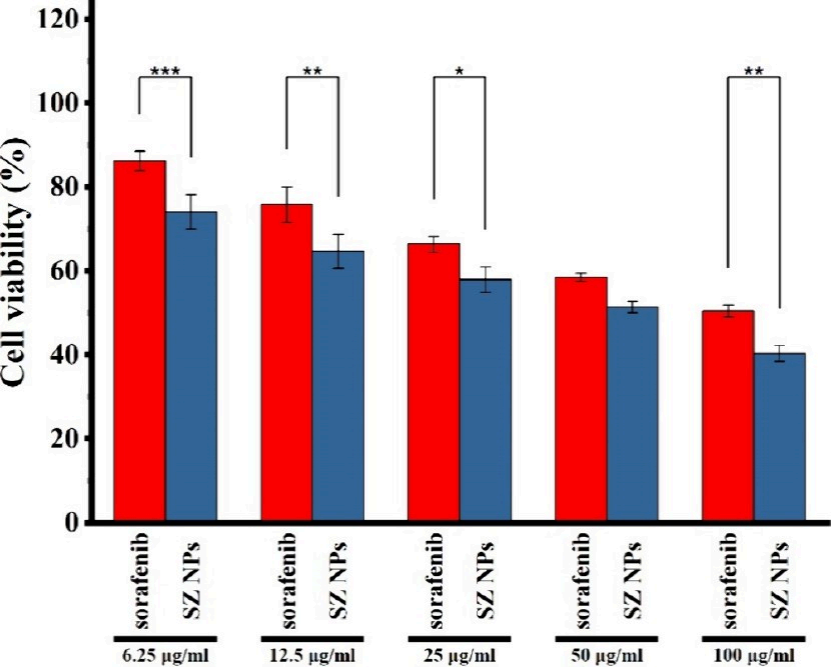
*In vitro* cytotoxicity assessment. Cell viability of HepG2 cells following a 48-h incubation with free sorafenib and SZ NPs at different concentrations (100, 50, 25, 12.5, 6.25 μg/mL).

### 3.6 Apoptosis Analysis

Inducing apoptosis is critical in cancer therapy as it provides opportunities to inhibit tumor growth, boost treatment effectiveness, and prevent metastasis.^60^ In this study, SZ NPs-induced apoptosis in HepG2 cells following a 24-h treatment was investigated using Annexin V-FITC/PI staining. As displayed in Figure 14, treatment with 12.5 and 25 μg/mL of SZ NPs led to apoptosis rates of 10.7% and 15.9%, respectively, indicating that the apoptotic effect of the SZ NPs was not noticeable at lower doses. Nevertheless, elevation of the concentration to 50, 100, and 250 μg/mL significantly promoted apoptosis induction to 21.5%, 47.9%, and 95.4%, respectively. In contrast, the untreated HepG2 cells in the control group revealed a high viability rate of 98.08% (Figure 14). These findings demonstrate the efficacy of SZ NPs in promoting apoptosis in HepG2 cells. The observed enhancement in apoptosis induction can be attributed to the effective delivery of sorafenib facilitated by the ZIF-8 NPs. This accords with Mi et al.’s results, which found that the apoptosis rate in HepG2 cells exposed to Dihydromyricetin@ZIF-8 for 24 h was 56.07 ± 1.27%.^61^

**Figure 14:**
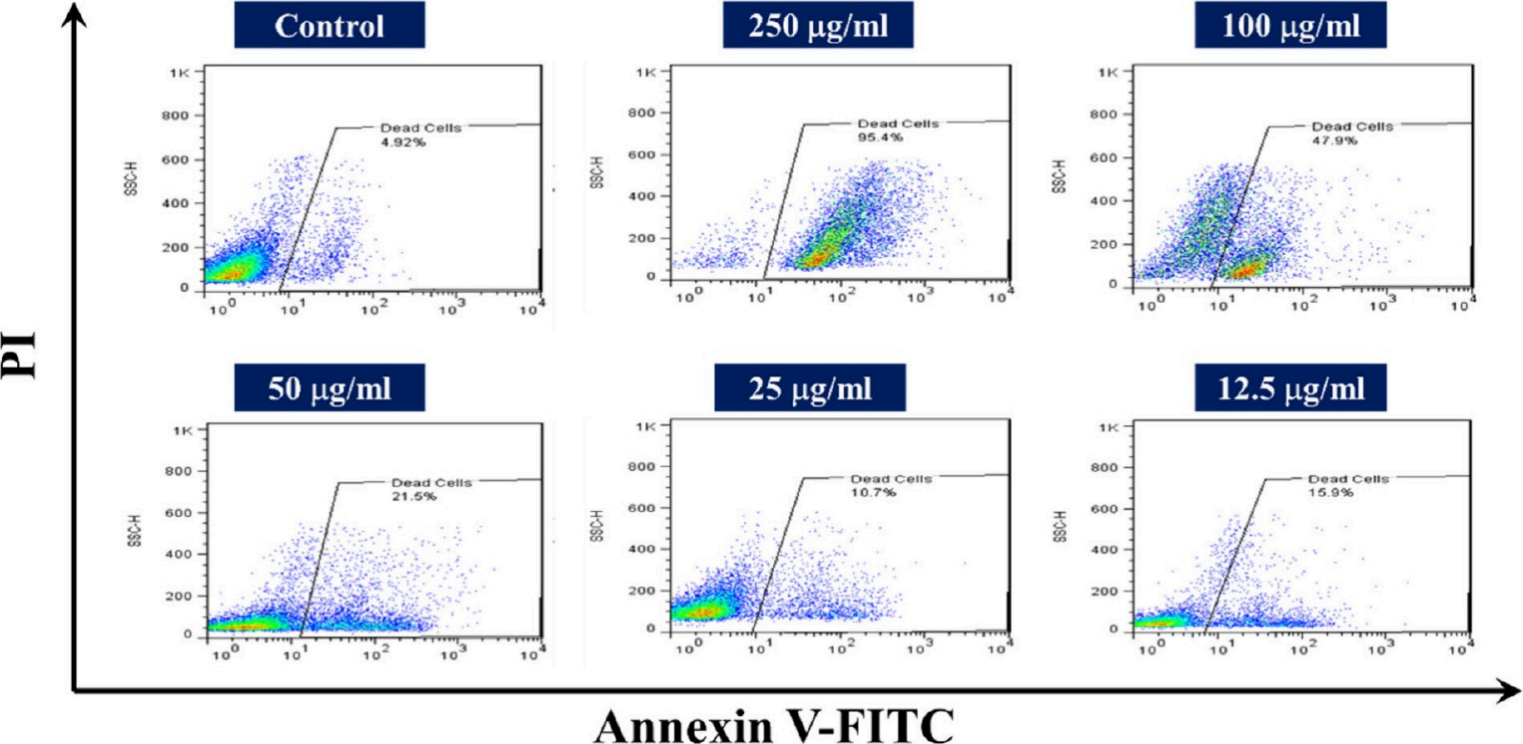
Induction of apoptosis. Apoptosis images of HepG2 cells treated with SZ NPs at different concentrations (12.5–250 μg/mL) compared to control untreated cells.

## 4 Conclusion

This study presented an innovative approach for oral delivery of sorafenib by encapsulating sorafenib-loaded ZIF-8 NPs into aMPs using a microfluidics method. More specifically, the synthesized SZ NPs, manifesting a sorafenib entrapment efficiency of 76.23%, were encapsulated into pH-responsive aMPs using a single-step droplet microfluidic technique based on a water-in-oil emulsion. This composite delivery system prevented premature release of sorafenib within the harsh conditions of the gastrointestinal tract; however, at pH ≥ 5.7, the drug-loaded ZIF-8 was efficiently released from the microcapsules. The demonstrated superior cytotoxicity of SZ NPs against HepG2 cell lines compared to free sorafenib verified the therapeutic potential of this nanodelivery system for HCC treatment. The boosted anticancer efficacy of the produced NPs, surpassing that of the free drug, corroborated the findings of the cytotoxicity assessments. This approach well indicated the feasibility of fabricating customized nano-in-microparticles capable of encapsulating a wide range of chemotherapeutics, including peptide or protein-based anticancer drugs. However, to comprehensively validate its biomedical applicability, further systematic evaluation of the carrier’s biocompatibility using normal human cell lines, along with *in vivo* investigations, is required. Such studies will be essential to confirm its pharmacokinetic profile, improved bioavailability, and antitumor efficacy, thereby providing deeper insights into its therapeutic potential and long-term safety for oral chemotherapy in HCC.

## Supplementary Material



## Notes

Fluorescent microscopy images; setup of the microfluidic chip and final nano-in-microparticles; summary of fabrication approaches (PDF)
