# Supplementary material for: Microfluidic Encapsulation of Sorafenib-Loaded ZIF‑8 Nanoparticles in pH-Responsive Alginate Microparticles for Oral Chemotherapy of Hepatocellular Carcinoma
Source: ACS Appl Bio Mater. 2026 Jan 28;9(4):1859–73. doi: 10.1021/acsabm.5c01270 (PMC12914634; doi:10.1021/acsabm.5c01270)
Supplement: Supplementary file 1 [file mt5c01270_si_001.pdf]

"Supporting Information"

Microfluidic Encapsulation of Sorafenib-loaded ZIF-8 Nanoparticles in a pH-Responsive Alginate Microparticles for Oral Chemotherapy of Hepatocellular Carcinoma

Mojdeh Mirshafiei[⊥1], Zahra Mahmoudi[⊥*2], Mehdi Mehrpouya[3,4] , Mahdi Mahmoudi[3,4], Masoud Rezaeian[5], Mona Navaei-Nigjeh[6,7], Zahra Katoli, [7,8] ,Lobat Tayebi[9*]

[1] Department of Biotechnology, School of Chemical Engineering, College of Engineering, University of Tehran, Tehran, Iran, mojdeh.mirshafiei@gmail.com

[2] Department of Medical Biotechnology, School of Biotechnology, College of Science, University of Tehran, Tehran, Iran, l.mahmoodi@ut.ac.ir

[3] School of Energy Engineering and Sustainable Resources, College of Interdisciplinary Science and Technology, University of Tehran, Tehran, Iran, mehrpoya@ut.ac.ir

[4] Hydrogen and Fuel Cell Laboratory, College of Interdisciplinary Science and Technology, University of Tehran, Tehran, Iran, mehrpoya@ut.ac.ir, mahdimahmoudi96@gmail.com

[5] Department of Chemical and Biological Engineering, The University of British Columbia, 2360 E Mall, Vancouver, BC V6T 1Z3, Canada, masoudrezaeian7528@gmail.com

[6] Department of Pharmaceutical Biomaterials and Medical Biomaterials Research Center, Faculty of Pharmacy, Tehran University of Medical Science (TUMS), Tehran, Iran, mnavaei@sina.tums.ac.ir

[7] Pharmaceutical Science Research Center (PSRC), Tehran University of Medical Science (TUMS), Tehran, Iran.

[8] Department of Life Science Engineering, Faculty of New Science & Technologies, University of Tehran, Tehran, Iran, zahrakatoli@yahoo.com

[9] Institute for Engineering in Medicine, Health, & Human Performance (EnMed), Batten College of Engineering and Technology, Old Dominion University, Norfolk, VA, 23529, USA, Ltayebi@odu.edu

⊥ These two authors contributed to the manuscript equally

Corresponding Authors: Zahra Mahmoudi, Lobat Tayebi (l.mahmoodi@ut.ac.ir)

(Ltayebi@odu.edu)

(Figure S1): Rhodamine B labeled ZIF-8 NPs encapsulated in alginate microparticles under brightfield (A), and fluorescent (B) microscopes.

To better visualize NPs encapsulated in alginate microparticles, we incorporate rhodamine B, a generic fluorophore (83689 Sigma-Aldrich), into ZIF8 NPs. To do this, rhodamine B was incorporated into the synthesis of ZIF-8 nanoparticles.

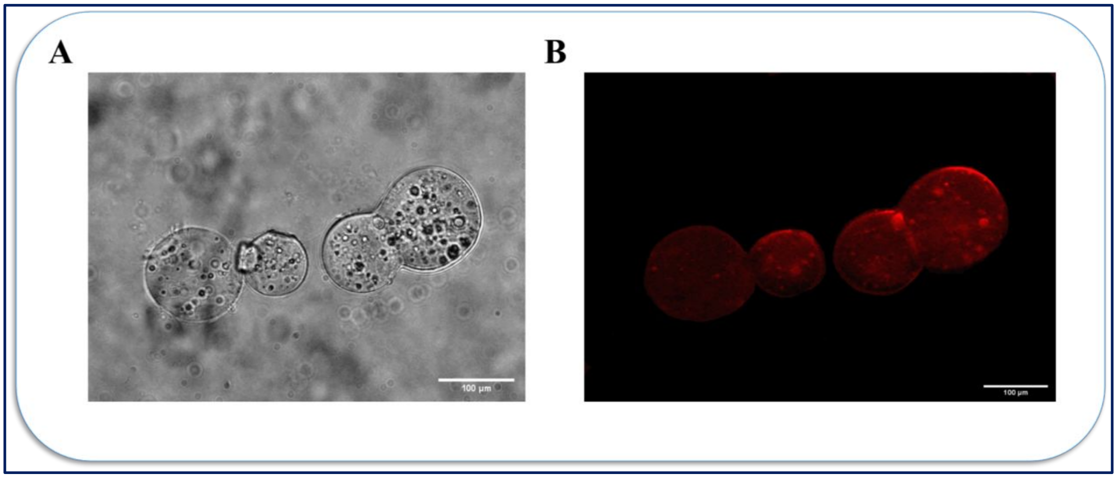

(Figure S2): Images related to the microfluidics setup (left image), and the images captured with bright field microscopy of alginate microparticles loaded with sorafenib-loaded ZIF-8 nanoparticles.

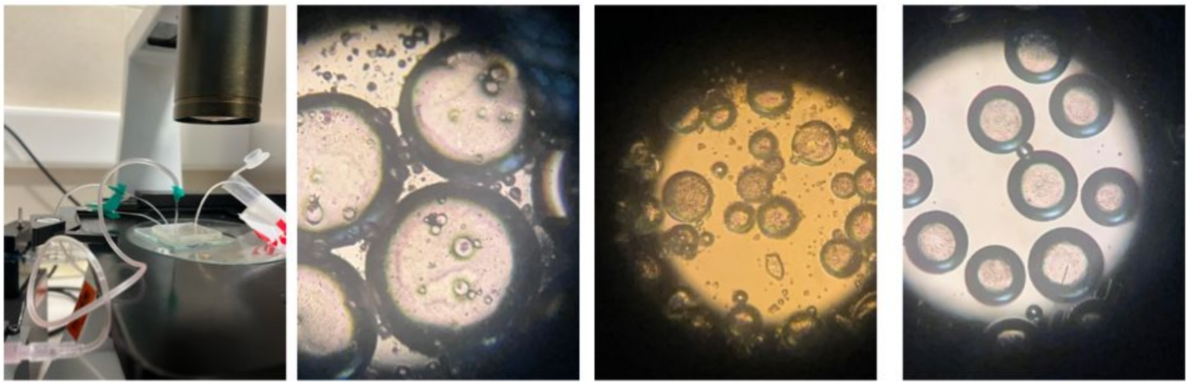

(Table S1). Common fabrication approaches for alginate-based nano-in-micro encapsulation systems.

Fabrication Method	Particle Uniformity and Size	Advantages	Limitations	Example Platforms	Ref
Ionic Gelation	Low–moderate uniformity; broad size distribution due to diffusion-based crosslinking.	Simple, inexpensive, and scalable; mild aqueous conditions preserve drug/NP activity.	Broad size distribution; limited control of size and shape; diffusion-limited crosslinking may cause gradients; prone to aggregation.	cabazitaxel (CAB)-loaded poly(alkyl cyanoacrylate) (PACA) @alginate[1]	[1]
Electrospray	Good uniformity; tunable sizes; can produce multi-core structures.	Excellent control over particle size and uniformity; scalable; single-step; mild for cargos, NP and cell-bioactive factor loading	Requires optimization of voltage and flow rate for NP distribution and droplet formation; limited to low-viscosity solutions; voltage sensitivity may cause jet instability; conductivity of solution affects process; potential aggregation risk.	(bFGF encapsulated PLGA)/ZIF-8@ sodium alginate microspheres [2]	[2-4]
Extrusion / dripping	Moderate uniformity; Broad size distribution (Depending on needle or nozzle size, or dripping height variability).	Simple, scalable, inexpensive; gentle processing; minimal equipment.	Limited control over size; particle shape variability; clogging risks.	ZIF-8/calcium alginate[5]	[5]
Microfluidic	Excellent uniformity; tunable size; spherical morphology.	Highly monodisperse particles; precise control of composition and structure; high reproducibility; gentle encapsulation.	Low per-device throughput; requires microfluidic setup; channel clogging risk.	Yolk/shell ZIF-8/alginate[6] & Current study	[6]

References

(1) Fleten, K. G.; Hyldbakk, A.; Einen, C.; Benjakul, S.; Strand, B. L.; Davies, C. L.; Morch, Y.; Flatmark, K. Alginate Microsphere Encapsulation of Drug-Loaded Nanoparticles: A Novel Strategy for Intraperitoneal Drug Delivery. *Mar. Drugs* **2022**, *20* (12), 744. DOI: 10.3390/md20120744.
(2) Song, S.; Li, Z.; Li, J.; Liu, Y.; Li, Z.; Wang, P.; Huang, J. Electrospray Nano-Micro Composite Sodium Alginate Microspheres with Shape-Adaptive, Antibacterial, and Angiogenic Abilities for Infected Wound Healing. *ACS Appl. Mater. Interfaces* **2024**, *16* (22), 28147-28161. DOI: 10.1021/acsami.4c03655.
(3) Li, H.; Yu, H.; Su, W.; Wang, H.; Tan, M. Tuning the Microstructures of Electrospray Multicore Alginate Microspheres for the Enhanced Delivery of Astaxanthin. *ACS Omega* **2023**, *8* (44), 41537-41547. DOI: 10.1021/acsomega.3c05542.
(4) Zhang, C.; Wang, X.; Xiao, M.; Ma, J.; Qu, Y.; Zou, L.; Zhang, J. Nano-in-micro alginate/chitosan hydrogel via electrospray technology for orally curcumin delivery to effectively alleviate ulcerative colitis. *Mater. Des.* **2022**, *221*, 110894.
(5) Song, Y.; Wang, N.; Yang, L.-y.; Wang, Y. g.; Yu, D.; Ouyang, X.-k. Facile fabrication of ZIF-8/calcium alginate microparticles for highly efficient adsorption of Pb (II) from aqueous solutions. *Ind. Eng. Chem. Res.* **2019**, *58* (16), 6394-6401.
(6) Liu, W.; Zhao, Y.; Zeng, C.; Wang, C.; Serra, C. A.; Zhang, L. Microfluidic preparation of yolk/shell ZIF-8/alginate hybrid microcapsules from Pickering emulsion. *Chem. Eng. J.* **2017**, *307*, 408-417.
